# Are Clowns Good for Everyone? The Influence of Trait Cheerfulness on Emotional Reactions to a Hospital Clown Intervention

**DOI:** 10.3389/fpsyg.2017.01973

**Published:** 2017-11-13

**Authors:** Sarah Auerbach

**Affiliations:** Personality and Assessment, Department of Psychology, University of Zurich, Zürich, Switzerland

**Keywords:** trait cheerfulness, Facial Action Coding System, Duchenne smile, hospital clown, amusement, transcendence

## Abstract

Trait cheerfulness predicts individual differences in experiences and behavioral responses in various humor experiments and settings. The present study is the first to investigate whether trait cheerfulness also influences the impact of a hospital clown intervention on the emotional state of patients. Forty-two adults received a clown visit in a rehabilitation center and rated their emotional state and trait cheerfulness afterward. Facial expressions of patients during the clown visit were coded with the *Facial Action Coding System*. Looking at the total sample, the hospital clown intervention elicited more frequent facial expressions of genuine enjoyment (Duchenne smiles) than other smiles (Non-Duchenne smiles), and more Duchenne smiles went along with more perceived funniness, a higher level of global positive feelings and transcendence. This supports the notion that overall, hospital clown interventions are beneficial for patients. However, when considering individual differences in the receptiveness to humor, results confirmed that high trait cheerful patients showed more Duchenne smiles than low trait cheerful patients (with no difference in Non-Duchenne smiles), and reported a higher level of positive emotions than low trait cheerful individuals. In summary, although hospital clown interventions on average successfully raise the patients’ level of positive emotions, not all patients in hospitals are equally susceptible to respond to humor with amusement, and thus do not equally benefit from a hospital clown intervention. Implications for research and practitioners are discussed.

## Introduction

Whereas in some situations all people behave more or less in the same way, in other situations, individual differences co-determine people’s actions and reactions. Research in the field of positive psychology has shown that in situations designed to promote happiness and well-being, the fit between a person’s personality and the type of activity is in part responsible for its success (Schueller, 2012; Senf and Liau, 2013). The present study focuses on hospital clown interventions^1^, which aim at bringing positive experiences to ailing patients. Hospital clown interventions have been described to “represent a particular way of using humor in order to promote people’s well-being” (Dionigi et al., 2012, p. 1). The idea goes back to Freud (1960), who described humor as a tool that allows the individual to face adversity. In a situation normally associated with negative emotions (such as a hospital stay), humor can help the individual to cope with the situation by providing an alternative perspective on the situation. Although the art of clowning does not solely consist of humor (e.g., Peacock, 2009), humor has frequently been characterized as the main component of hospital clowning (Dionigi et al., 2012), and thus hospital clown interventions have been defined as humorous interventions^2^ (Ruch and Hofmann, 2017).

Although to date, hospital clown interventions are widely used in hospitals, nursing homes and other care facilities, and research has shown some positive effects for patients (see Effects of Hospital Clown Interventions on Individuals), no study has investigated whether these humorous interventions are beneficial for all recipients, or whether some groups of individuals benefit more than others (only age or gender differences were tested so far; e.g., Fernandes and Arriaga, 2010; Vagnoli et al., 2010). Hence, no research is available on whether individual differences influence the effects of a hospital clown intervention on the emotional reactions of patients.

### Individual Differences in Emotional Reactions to Humor

Research on personality and humor demonstrates that people habitually differ in the way they cognitively evaluate humorous stimuli (Ruch and Hehl, 2007), use and communicate humor in everyday life (Craik et al., 1996; Fox et al., 2016), and emotionally respond to humor (Ruch, 2007; Platt et al., 2013; Ruch et al., 2015). The predominant emotional reaction to humor was labeled *exhilaration*^3^ (or amusement), which in classifications of emotions is defined as a facet of joy (Ruch, 1993). One personality trait in particular, *trait cheerfulness*, has been studied in a variety of humor experiments and settings as a stable disposition for cheerful mood states and the easiness with which amusement is induced. Trait cheerfulness is characterized by a prevalence of cheerful mood, a low threshold for smiling and laughter, a composed view of adverse life circumstances, a broad range of active elicitors of cheerfulness and smiling and laughter, and a generally cheerful interaction style (Ruch et al., 1996). Together with trait seriousness and trait bad mood, it forms the temperamental basis for the sense of humor (Ruch and Carrell, 1998). Trait cheerfulness can be classified into the higher-order dimension of extraversion (Carretero-Dios et al., 2014), but has a higher specificity in predicting the intensity of amusement in response to humor than extraversion (Ruch, 1997). Ruch et al. (2011) postulated five relationships between trait cheerfulness and a cheerful state: high trait cheerful individuals have a lower threshold, a higher intensity, a longer duration, a higher robustness of cheerful mood (even when facing adversity), and a faster mood recovery (after a mood alteration to the negative) than low trait cheerful individuals. These postulates were tested in various experiments and contexts using subjective as well as objective methods, such as the observation of facial signs, to infer on the emotional state (for an overview see Ruch and Hofmann, 2012).

The universal facial expression of enjoyable emotions is smiling (Ekman, 2003). Research has repeatedly shown that there are different types of smiles, but especially one type (*Duchenne smile*) is a valid indicator of genuine enjoyment (Ekman et al., 1990; Sauter et al., 2013). It is characterized by the joint and timely corresponding contraction of the zygomatic major muscle (pulling the lip corners up) and the lateral part of the orbicularis oculi muscle (contracting the region around the eye producing crow’s feet). Other types of smiles occur in situations without genuinely felt enjoyment (*Non-Duchenne smiles*). These types of smiles are present, for example, when individuals mask a negative emotional state (masking smile) or smile when nothing much is felt (phony smile) but individuals attempt to appear as if positive emotions are felt (Ekman and Friesen, 1982; Harris and Alvarado, 2005). The different types of smiles can be assessed with an objective and reliable technique for coding observable facial actions, the *Facial Action Coding System* (FACS; Ekman et al., 2002a), which enables coding the frequency, intensity, timing, duration, laterality and symmetry of 44 different action units. In a series of studies the FACS was used as an objective measure of amusement to demonstrate the influence of trait cheerfulness on the emotional reaction to humorous stimuli. For example, during an interaction with a clowning experimenter, individuals high in trait cheerfulness showed more frequent, more intense and longer lasting signs of facial amusement (Duchenne smiling and laughter^4^) than individuals low in trait cheerfulness (Ruch, 1997). When high trait cheerful individuals saw their own distorted photographs as a surprise, they showed more frequent Duchenne smiling and laughter than low trait cheerful individuals (Beermann and Ruch, 2011). When a virtual companion was present during a funny film, high trait cheerful individuals had higher frequencies of Duchenne laughter than low trait cheerful individuals (Hofmann et al., 2015).

### Effects of Hospital Clown Interventions on Individuals

To date, a few studies have evaluated hospital clown interventions and have consequently shown that hospital clown interventions can have a beneficial effect on patients (mostly children). For example, studies found a reduction of preoperative anxiety and worries in children undergoing medical procedures when interacting with a clown pair compared to a control group without a clown visit (e.g., Golan et al., 2009; Fernandes and Arriaga, 2010; Vagnoli et al., 2010). Regarding changes in positive states after interacting with hospital clowns, one study found an increase in self-rated positive affect in children (Fernandes and Arriaga, 2010), and another study found an increase in self- and parent reported well-being (Pinquart et al., 2011).

Only two studies have examined the positive emotions elicited by hospital clowns in individuals in more detail. Auerbach et al. (2014) developed and tested the *29 Clown Emotion List* (CLEM-29), which is a collection of single adjectives and short phrases, but can be reduced to four factors: amusement, transcendence, unease and arousal. The factor of amusement merges a variety of positive humor-related states including a calmer cheerfulness and a more aroused hilarity. Transcendence was defined according to its non-religious connotation as the feeling of being uplifted and surpassing the ordinary. It includes positive feelings induced by clowns such as feeling privileged, appreciated, connected to the clown and elevated. The negative factor of unease consisted of negative feelings induced by clowns (e.g., threatened, fearful, confused). Ratings of the factor of arousal relate to different states of arousal, in which positive loadings (touched and speechless) refer to a calm state, that is, low arousal, whereas negative loadings (overexcited and *schadenfreude*) refer to a more heightened arousal. Studies that used the CLEM-29 showed that individuals watching videos of (Auerbach et al., 2014) and patients interacting with (Auerbach et al., 2016) a hospital clown reported a higher level of amusement compared to individuals who watched or experienced a nurse intervention. Furthermore, in both samples a combination of amusement and transcendence best predicted the total amount of positive affect after a hospital clown intervention. The authors concluded that a hospital clown intervention induces not only the typical humor reaction in recipients (amusement), but also adds a unique quality to the clown-patient interaction (transcendence).

In summary, previous research has provided evidence that hospital clown interventions are a suitable method to enhance the emotional state of individuals. The studies used subjective assessment tools, either self-reports or external reports of the key variables. So, the next step in a comprehensive evaluation of hospital clown interventions is to validate the subjectively assessed state of patients during the clown intervention by including observable signs of non-verbal behavior. Research (e.g., Ruch and Hofmann, 2012) has shown that humorous stimuli successfully generate facial amusement in various experiments if the subjects experience amusement (objective and subjective markers of amusement are typically moderately related; Ruch, 1995). Platt et al. (2013) showed that of the 16 enjoyable emotions proposed by Ekman (2003), amusement was one facet of joy that went along with both Duchenne smiles and Duchenne laughter. It is assumed that amusement will be the enjoyable emotion most elicited by hospital clown interventions, going along with Duchenne smiles and laughter.

Another still unnoted issue in evaluations of hospital clown interventions is the personality influence. Taking into account the trait cheerfulness model and its empirical evidence (Ruch and Hofmann, 2012), it can be assumed that high trait cheerful individuals benefit more from the intervention (i.e., more positive emotions) than low trait cheerful individuals.

### Aims and Hypotheses

The present study aims to contribute to a better understanding of hospital clown interventions in three ways: the investigation of patient’s facial signs of enjoyment during an interaction with a hospital clown, its relationship to their subjective states, and the replication of the theory of trait cheerfulness as predictor of the emotional reaction of patients to humorous stimuli. The first hypothesis is that the hospital clown intervention on average elicits Duchenne smiles more often than Non-Duchenne smiles. The second hypothesis is that higher frequencies of Duchenne smiles are associated with higher levels of a positive experience, and lower levels of a negative one, whereas higher frequencies of Non-Duchenne smiles are associated with lower levels of positive experiences, and higher levels of negative ones. The third hypothesis is that high trait cheerful individuals show more Duchenne smiles and less Non-Duchenne smiles, and simultaneously report higher levels of positive emotions than low trait cheerful individuals.

## Materials and Methods

### Sample

The sample consisted of *N* = 42 adult German speaking patients from a physical rehabilitation center (81% male), and was a convenient sample. Patients suffered from paraplegia, amputations, or other multiple injuries. The age of patients ranged from 19 to 75 years (*M* = 45.36, *SD* = 16.56). Inclusion criteria were age 18 or older, voluntary participation, not bedridden, and being cognitively and physically able to participate in the study. Patients were filmed during the study, and videos of a subsample of 26 patients could be used for coding facial actions.

### Instruments

The standard trait version of the *State-Trait-Cheerfulness Inventory* (STCI-T < 60 >; Ruch et al., 1996) consists of 60 items to reliably and validly assess trait cheerfulness, trait seriousness and trait bad mood. To compose the *trait cheerfulness* scale in the current study, eight items were selected representing the facets of a low threshold for smiling and laughter and a generally cheerful interaction style (hilarity^5^; e.g., “I am a merry person”). The answer format is a four-point Likert-scale ranging from 1 (strongly disagree) to 4 (strongly agree) and Cronbachs alpha was 0.87.

The *29 Clown Emotion List* (CLEM-29; Auerbach et al., 2014) is a list of 29 adjectives and short phrases assessing emotional states in the context of clowning. Participants rate their current state on a 7-point Likert scale ranging from 1 (=not at all) to 7 (=very strongly). As the sample size in the present study is too small to test the hypotheses with all single ratings, factor scores were used instead (transcendence, uneasiness, amusement, and arousal; the procedure is described in detail in Auerbach et al., 2016), which are sensitive enough to capture changes in clown-induced emotional states (Auerbach et al., 2014).

The *Hospital Study Evaluation Form* (HSEF; Auerbach et al., 2016) contains 22 single ratings, of which seven ratings concern the stay in the care facility (HSEF-General; e.g., quality of meals, care) and the evaluation of the hospital clown intervention (HSEF-Current; e.g., global positive and negative feelings during the situation). The answer format is a 7-point Likert scale. A second set of 15 single ratings (HSEF-Preferences), which are related to patients’ general preferences for clowns, was given to patients at the end of the study (e.g., general liking of clowns; 5-point Likert scale).

### Procedure

Prior to the study, the local ethics committee approved the study. Consent forms were handed out before and after the experiment. The core of the current study was a surprise visit from a hospital clown pair. The study took place in a separate room in the rehabilitation center, and the procedure was highly standardized. Patients were recruited with the cover story that they were going to participate in an evaluation of patient satisfaction in hospitals. They were also told that a staff member would conduct a routine assessment, which they were to evaluate afterward. Two patients participated in each trial. Patients first filled out the HSEF-General, followed by a baseline assessment of emotional states (CLEM-29, HSEF-Current). Afterward, the clown intervention of a predetermined length took place (Min = 4.00, Max = 8.85, *M* = 6.65, *SD* = 1.17). It consisted of a semi-standardized performance of a hospital clown pair (one male clown with 17 years of experience, and one female clown with 16 years of experience), aiming at the induction of a positive emotional state in the patients. The same clowns performed in all trials, and used the same roles, clothes and make-up. They worked according to a script and did the same performance (same punchlines) in every trial. They were instructed to limit the length of the interaction to about 5–8 min. Both wore a red nose. The male clown carried a ukulele, wore a Doctor-like jacket. The female clown wore a yellow dirndl dress with yellow socks, a pink blouse. She had an abnormally large handbag in one hand filled with requisites; e.g., a pig nose that makes a farting sound when squeezed, and a thimble, used to demonstrate a magic trick together. The clown pair behaved like Auguste and Whiteface: the female clown was more dominant, slightly aggressive, bossy and pompous, while the male clown was the foolish, clumsy and more sensitive partner. After the clowns left the room, patients filled out the state measures (CLEM-29, HSEF-Current). Patients subsequently were debriefed about the real aim of the study (to investigate emotional reactions to hospital clowns) and asked not to disclose the use of clowns to other patients until the study was completed. For the last step of the study, they filled out the trait measures (STCI-T < 60 >, HSEF-Preferences).

Full color, digital format films with a close-up view of the patients’ face were recorded. To be able to code the same clown-patient interactions for all subjects, ten standardized scenes occurring in all trials (about 10–20 s long) were extracted, each containing a studied punch line produced by the clowns followed by the reaction of patients. A certified FACS coder^6^ coded the resulting 260 observations (26 patients with 10 scenes each) with the help of the FACS (Ekman et al., 2002a). A Duchenne smile was defined as a symmetric and timely coincidental movement of the orbicularis oculi muscle around the eye (AU6) and zygomatic major muscle at the corners of the mouth (AU12). It could be accompanied by a tightening of the eyelids (AU7) and mouth opening (AU25, AU26, AU27), but no other action unit^7^ (Ekman and Friesen, 1982). The Non-Duchenne smile was defined as AU12 alone, or AU12 plus further action units that are associated with negative feelings (Ekman et al., 2002b). Laughter vocalizations were coded using one of four codes: “single unvoiced (ch),” “single voiced (ha),” “multiple unvoiced (ch ch ch),” or “multiple voiced (ha, ha, ha).”

## Results

Three scores were built for use in the analyses. As they were sum scores over ten different standardized scenes during the interaction between the clowns and a patient, Cronbach’s alpha was calculated for each score as a measure of the homogeneity of behaviors during the ten scenes. A frequency score for enjoyment smiles was built by summing up all Duchenne smiles in ten scenes, which showed high internal consistency (*α* = 0.80). A frequency score for Non-Duchenne smiles was built by summing up all Non-Duchenne smiles in the same ten scenes (*α* = 0.58). The Non-Duchenne smile category was more heterogeneous than the Duchenne smile one, as it comprised different types of Non-Duchenne smiles. A laughter score was built by summing up all four types of laughter vocalizations in ten standardized scenes (*α* = 0.75). All variables used in the analyses were normally distributed.

### Frequency of Different Types of Smiles

Patients on average smiled 8.92 times (*SD* = 3.76) during the 10 scenes. The percentage of Duchenne smiles among all smiles was 76.29%. The minimum was zero Duchenne smiles; the maximum was 16 (*M* = 6.81, *SD* = 3.74). The minimum of Non-Duchenne smiles was zero; the maximum was eight (*M* = 2.12, *SD* = 2.01). Patients on average laughed 3.58 times during the ten scenes (*SD* = 4.14) with a maximum of 15 laughter vocalizations. Thirty percent of patients did not produce any laughter vocalizations during the selected scenes.

### Relationship between Subjective and Objective Assessment

Negative affect after the clown visit was very low (*M* = 1.73, *SD* = 1.32), and positive affect was high (*M* = 5.12, *SD* = 1.5; scale from 1 to 7). Patients enjoyed participating in the study to a high extent (*M* = 4.24, *SD* = 0.77; scale from 1 to 5), and 81.5% stated that they felt better after the clown visit. The frequency of Duchenne smiles was positively correlated with funniness of the clown visit (*r* = 0.57, *p* < 0.01), global positive feelings (*r* = 0.46, *p* < 0.01), transcendence (*r* = 0.40, *p* < 0.05) and the joy of participating in the study (*r* = 0.43, *p* < 0.05), and negatively correlated with global negative feelings after the clown visit (*r* = -0.38, *p* < 0.05). The frequency of Non-Duchenne smiles was negatively correlated with the joy of participating in the study (*r* = -0.62, *p* < 0.01), transcendence (*r* = -0.59, *p* < 0.01), feeling better after the clown visit (*r* = -0.33), and positively correlated to unease (*r* = 0.34, both marginally not significant, *p* = 0.06). Laughter vocalizations were positively correlated with Duchenne smiles (*r* = 0.37, *p* < 0.05), transcendence (*r* = 0.46, *p* < 0.05), amusement (*r* = 0.44, *p* < 0.05), funniness of the clown visit (*r* = 0.46, *p* < 0.01), and feeling better after the clown visit (*r* = 0.41, *p* < 0.05).

### The Influence of Trait Cheerfulness

**FIGURE 1:**
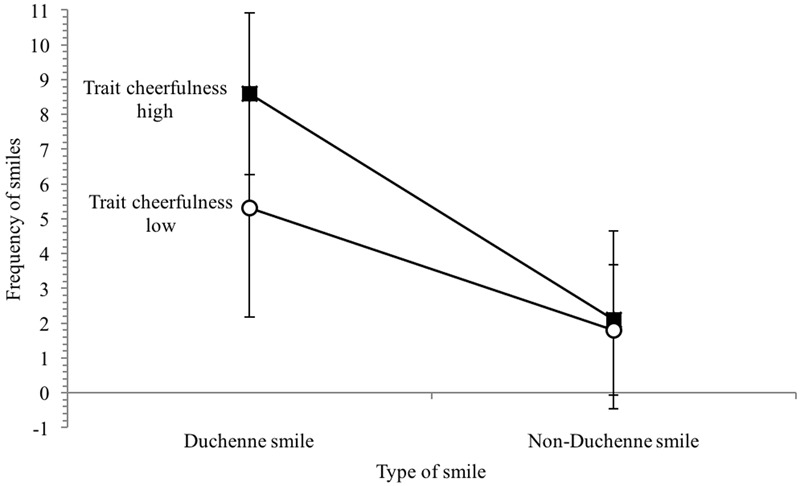
Frequencies of smiles during the hospital clown intervention for individuals high and low in trait cheerfulness.

Next, it was tested whether high trait cheerful individuals had higher levels of positive emotions during the clown intervention than low trait cheerful individuals. To build two groups of equal sizes, ten patients with the lowest scores were allocated to group 1 (low trait cheerful), and ten patients with the highest scores to group 2 (high trait cheerful). A 2 × 2 repeated measures ANOVA with trait cheerfulness (high vs. low) and type of smile (Duchenne smile vs. Non-Duchenne smile) was computed for the frequency of smiling. Results are displayed in**Figure 1**.

Patients showed more Duchenne smiles than Non-Duchenne smiles, *F*(1,18) = 31.58, *p* < 0.001, $\eta_{p}^{2}$ = 0.64, and high trait cheerful individuals smiled more frequently than low trait cheerful individuals, *F*(1,18) = 6.90, *p* < 0.05, $\eta_{p}^{2}$ = 0.28. The interaction just failed to be significant, *F*(1,18) = 2.84, *p* = 0.11. However, there was a numerical trend toward higher levels of Duchenne smiles in the high trait cheerful group (*M* = 8.60, *SD* = 2.32) than in the low trait cheerful group (*M* = 5.30, *SD* = 3.13). An independent samples *t*-test confirmed that the two groups significantly differed in their frequency of Duchenne smiles, *t*(18) = -2.68, *p* < 0.05. No difference was found for Non-Duchenne smiles, *t*(18) = -0.30, *p* = 0.77.

Individuals high in trait cheerfulness reported higher positive feelings, *t*(31) = -2.35, *p* < 0.05, higher funniness ratings of the clowns, *t*(31) = -2.82, *p* < 0.01, higher levels of transcendence (marginally not significant), *t*(28) = -1.75, *p* = 0.09, and a lower level of unease, *t*(28) = 3.09, *p* < 0.01, than individuals low in trait cheerfulness. The two groups did not differ in their general preference for clown performances, *t*(31) = -1.54, *p* = 0.14, and laughter, *t*(18) = -0.70, *p* = 0.49.

## Discussion

Humor interventions have been used frequently in research to increase happiness and lower depression in various settings and samples, including hospital clown interventions (for an overview see Ruch and Hofmann, 2017). One consistent finding stemming from humor research is that individuals habitually differ in their readiness to react with amusement to humorous stimuli (Ruch and Hofmann, 2012). However, this has never been tested in patients receiving a hospital clown visit. Hence, the present study was the first to investigate individual differences in the emotional state of patients in response to a hospital clown intervention, and to use the FACS as a comprehensive, reliable technique for the objective assessment of the patient’s emotions. This made it possible to distinguish between Duchenne smiles (genuine expressions of enjoyment) and other smiles in patients during clown-patient interactions.

First, the results confirmed that both types of smiles can occur during a humorous intervention (Harris and Alvarado, 2005), but eight out of ten smiles were Duchenne smiles, which is associated (and was positively correlated) with a positive emotional state (Ekman, 2003). In the present study, the facial expression of enjoyment was not only highly related to funniness ratings of the hospital clown performance indicating amusement (which replicated findings from humor research; Ruch, 1995, 1997), but also positively related to the felt level of transcendence in patients (extending humor research). Hence, the present study complements the work of other researchers and practitioners who stress that hospital clown interventions are not eliciting amusement, but contribute to the elicitation of other positive experiences. Patch Adams, one of the pioneers of hospital clowning, described the work of hospital clowns as a combination of humor and *love* (Adams, 2002). Kontos et al. (2017) found that clowns working with elderly care residents apply a mixture of humor and empathy. Linge (2012) interviewed children after a hospital clown intervention and concluded that a close connection between the clown and the recipients (*magical attachment*) is a core component of a (successful) clown-patient interaction. Hospital clown interventions apparently elicit feelings that go beyond the typical humor response, such as feelings of connection, liberation, appreciation or playfulness. In this sense, the present research validates studies using self-report measures (Auerbach et al., 2014, 2016), and strengthens the widespread assumption of practitioners and clown organizations (see Dionigi et al., 2012) that on average hospital clown interventions successfully create positive experiences and emotions for patients in need of care.

Second, another important, yet unanswered question was whether a hospital clown intervention is successful in eliciting a positive emotional state in all patients, or whether some groups of patients benefit more from the intervention than other groups. Derived from the theory of the temperamental basis of the sense of humor (Ruch et al., 1996), a trait could be identified that has been shown to be an important predictor for the emotional reaction to humorous stimuli repeatedly – trait cheerfulness (e.g., Ruch, 1997; Hofmann et al., 2015). The present study gives further validation to trait cheerfulness as predictor of positive emotions by demonstrating that a hospital clown intervention does not lead to high levels of amusement in all cases. Hence, not all patients benefit equally from the clown intervention. Clowns working in the field should always bear in mind that some patients do not want to be involved in a humorous and playful interaction, look for signs of refusal, and act accordingly. At the same time, the results can also be a justification for practitioners on a ‘bad day’ (e.g., in case their performance does not lead to the intended success, i.e., the patients do not smile or laugh). In fact, in many clown organizations hospital clown training includes interpersonal skills, the sensitization of the clowns to the current state of patients, and the appropriate handling of uncertainty and refusal (Dionigi et al., 2012), which seems even more important given the results presented here.

The present study has some limitations. First, only one clown pair was used. A next step could be to study possible interactions between high and low trait cheerful individuals and different kinds of clowns with different techniques (clown-person fit). The clown pair used in this study had a rather playful, interactive, hilarity-based style, while other clowns work in a more sensitive, insightful and composed way (Hofmann et al., 2014). Also, cultural differences in humor and clowning have not been studied here. Second, the sample was rather small and very heterogeneous with a wide age range and few females, which was due to the convenience sampling method. Also, only one physical rehabilitation center was included. Future research should collect larger samples more representative of hospitalized adult patients in different settings. Third, the situation was somewhat artificial – as patients were overtly filmed during the intervention – and the intervention was highly standardized, and other than in real life the subjects were committed to take part in the study. Results presented here might underestimate the true relationships between the behavior, subjective experience and personality of individuals. A next study should also aim to differentiate the different types of Non-Duchenne smiles and have a look at their correlations with different emotional states, while subjects are unobtrusively filmed. A recent study suggests that in spontaneous and unobserved situations, the emotional state of *Schadenfreude* goes along with the Duchenne smile, whereas in social situations (such as the openly filmed hospital clown intervention), subjects try to mask or suppress the expression of *Schadenfreude* (Hofmann and Ruch, 2015). It would be interesting to study the different types of smiles during a natural unobserved clown-patient interaction and during a social, observed situation, such as the one used in the present study. Fourth, although the main aim was to standardize the interactions between the clown pair and the patients, it is safe to say that not all trials were executed in exact the same manner. The clowns were instructed to perform as standardized as possible, but also as realistic as possible, meaning that in case the subject tried to interrupt the clown pair, they should not ignore him or her but react in a natural way before continuing with the scripted performance. After all, it was a real interaction between the clowns and the patients in a natural setting, and therefore not perfectly standardized. However, for the analyses only those scenes were chosen that occurred in every interaction in the same manner (same punchline), and thus the biasing effect on the results is expected to be rather small.

Despite the limitations, the results promote the use of hospital clown interventions for the enhancement of a positive emotional state in patients in need of care, but also point out the relevance of accounting for individual differences in recipients of the interventions. It is much to be hoped that this will stimulate future studies in that other researchers also combine objective and subjective assessment methods to get a clearer picture of the variety and uniqueness of emotional responses of patients during a hospital clown intervention. Furthermore, this knowledge can be used by organizations that train clowns to raise the awareness of signs that help explaining the success and failure of hospital clown interventions in their work in hospitals to prevent unwanted side effects such as the induction of negative emotions and rejection. Research demonstrates that emotional expressivity may be a reliable sign of cooperative tendency in humans (Schug et al., 2010), indicating that clowns should watch out for facial signs of emotions in patients to find out whether they want to cooperate (and thus join the game). These days, many clown organizations already include a sensitive, careful and responsible approach in the interaction with patients in their curriculum, emphasizing to always pay attention to the emotional impact of their visit to patients (Dionigi et al., 2012). Clown organizations could go one step further and specifically include the recognition and interpretation of facial expressions of individuals into their training programs.

## Ethics Statement

This study was carried out in accordance with the recommendations of “Swiss Psychological Association”; and the Ethics Committee of the Department of Psychology, University of Zurich, with written informed consent from all subjects. All subjects gave written informed consent in accordance with the Declaration of Helsinki.

## Author Contributions

SA conceived and designed the work and was also involved in data collection, data analysis and interpretation, drafting the article, critical revision of the article, and final approval of the published version.

## Conflict of Interest Statement

The author declares that the research was conducted in the absence of any commercial or financial relationships that could be construed as a potential conflict of interest. The author and handling Editor declared their shared affiliation, and the handling Editor states that the process nevertheless met the standards of a fair and objective review.

## Footnotes

1 Also referred to as clown therapy, clown visit, medical clowning, or clown care.

2 Ruch and Hofmann (2017) argue that there are different types of humor interventions (such as self-administered individual humor interventions stemming from a positive psychology tradition, humor training programs to strengthen an individuals’ level of the sense of humor, or hospital clown interventions).

3 Exhilaration has been defined as the main emotional response to humor and denotes either the process of making cheerful or the temporary rise and fall of a cheerful state. To exhilarate in this sense means to make cheerful, or to amuse (Ruch, 1993).

4 Duchenne laughter typically occurs at higher levels of reported amusement, while Duchenne smiling occurs at lower levels of reported amusement (Ruch, 1993).

5 The present study addresses emotional reactions to clowns, which are a trigger of hilarity. Hence, only items representing facets of hilarity were selected to compose trait cheerfulness.

6 The coder proved high reliability (agreement index = 0.78) scoring video material from real interactions in the FACS final test.

7 In few cases, high intensity combinations of AU12 and AU6 (intensity scores of D-E on a scale from A – E) during apex were accompanied by nose wrinkling (AU9) and/or eyebrow-lowering frowning (AU4). Following Hofmann (2014), these smiles were classified as high intensity Duchenne smiles. In the offset of the AU12, few patients pulled down their lip corners (AU15) or slightly pressed their lips together (AU24). There was no time overlap with the apexes of the AU12 and thus they were seen as regulatory mechanism (Ekman et al., 2002b).
